# Factors associated with a clinically relevant reduction in menopausal symptoms of a standardized acupuncture approach for women with bothersome menopausal symptoms

**DOI:** 10.1186/s12906-021-03208-2

**Published:** 2021-01-13

**Authors:** Frans Boch Waldorff, Christine Winther Bang, Volkert Siersma, John Brodersen, Kamma Sundgaard Lund

**Affiliations:** 1 The Research Unit for General Practice and Section of General Practice, Department of Public Health, University of Copenhagen, Copenhagen, Denmark; 2 Department of Public Health, University of Southern Denmark, Research Unit of General Practice, Odense, Denmark; 3 Primary Health Care Research Unit, Region Zealand, Sorø, Denmark

**Keywords:** Acupuncture, Clinical trials, Primary care, Menopause

## Abstract

**Background:**

Little is known about factors associated with a clinically relevant reduction in menopausal symptoms through a brief acupuncture approach for women with moderate-to-severe menopausal symptoms.

**Methods:**

Post hoc analysis of a randomized controlled trial where participants were allocated to early versus late standardized acupuncture. Both the early group and the late group are included in this study. The late group got an identical intervention parallel staged by 6 weeks. By means of the relative importance, the effect was evaluated for both early versus late women with a 6-week follow-up. We included four symptom subscales from the validated MenoScores Questionnaire: hot flushes, day and night sweats, general sweating, menopausal-specific sleeping problems, as well as an overall score, which is the sum of the four outcomes in the analysis**.**

**Results:**

67 women with moderate to severe menopausal symptoms were included of whom 52 (77.6%) experienced a clinically relevant reduction in any of the four surveyed symptom subscales or overall score. 48 (71.6%) women experienced a clinically relevant reduction in any of the vasomotor symptom subscales: hot flushes, day and night sweats, general sweating.

Women with vocational education were most likely to experience improvement compared to women with higher education. Beyond education, other factors of some importance for a clinically relevant reduction were no alcohol consumption, two or more births and urinary incontinence.

**Conclusions:**

Level of education was the most consistent factor associated with improvement. Beyond education, other factors of some importance were no alcohol consumption, two or more births and urinary incontinence.

**Trial registration:**

This study was registered in ClinicalTrials.gov at April 21, 2016. The registration number is NCT02746497.

## Background

Menopausal symptoms are common and may affect quality of life, health status, and use of health services [1–3]. Most women experience menopause in their early 50s [4] and the symptoms last for 4–5 years on average [4–7]. The key symptom of menopause is hot flushes, which affects around 75% of menopausal women [5, 6, 8] and up to 10–20% report this symptom as very bothersome [5]. Other frequent menopausal symptoms are sweating and sleeping problems [9].

Previously, we have reported that a standardized and brief acupuncture treatment produced a fast and clinically relevant reduction among women with moderate-to-severe hot flushes, day and night sweating, general sweating as well as sleeping problems [10] and that overall effect on menopause-relevant outcomes was sustained up to 21 weeks after treatment stopped [11]. However, the literature on determinants for the effect of acupuncture is sparse [12], which is important to provide guidance to both the women and the acupuncturist.

Therefore, the objective of this study was to investigate which factors are important for a clinically relevant reduction in menopausal symptoms in a standardized brief acupuncture approach for women with moderate-to-severe hot flushes.

## Methods

### Design and intervention

The study used data from a randomized trial evaluating the effectiveness of a standardized acupuncture intervention for women with moderate to severe menopausal symptoms [10]. We included healthy women aged 40–65 years experiencing moderate to severe menopausal symptoms evaluated by the hot flushes (HF) subscale from the MenoScores Questionnaire (MSQ) [9]. Women were excluded if they had undergone hysterectomy and/or bilateral oophorectomy, if they used other treatments for menopausal symptoms or treatments that might affect menopausal symptoms or if they had received acupuncture treatment within the past six months before enrolment. All participants were provided with oral and written information about the study and participation was voluntary, the participant could withdraw their consent at any time.

Women were randomly allocated to an early intervention (*n* = 36) and late intervention (*n* = 34) with a cross over at week 6. The intervention in both groups were identical but parallel staggered by 6 weeks. Women from both the early and late group are included in this study and we included allocation status as a confounder in the analysis.

The intervention consisted of one treatment with a needle retention time of 10 min every week for five week. The acupuncture was Western medical acupuncture (WMA) [13] and the predefined acupuncture points were: CV-3, CV-4, LR-8, SP-6, SP-9 (LR-8, SP-6 and SP-9 bilaterally). The acupuncturists were all GPs with on average 153 h of acupuncture education (range 80–300) and had practiced acupuncture for 14 years (range 4–38 years). All but one were educated in acupuncture by the Danish Society for Evidence-based Acupuncture (DSEA) or the Danish Medical Acupuncture Society. Further details on the intervention are available in our previously published paper [14].

### Assessments

The importance of a selection of factors was evaluated on four symptom subscales from the MSQ [9] – three vasomotor symptom (VMS) subscales: HF, day and night sweats (DNS), general sweating (GS), and menopausal-specific sleeping problems (MSSP) – as well as an overall score which is the sum of the four outcomes**.** The MSQ is a validated condition-specific patient-reported outcome measure consisting of 11 subscales of which the above four are most directly HF connected to menopause [9]. The scores of each of the symptom scales were related to a global item inquiring into whether the woman was bothered by menopausal symptoms. The difference in mean symptom score from ‘a lot’ on the global item to ‘quite a bit’ on the global item was considered a clinically relevant reduction. For each of the symptom scales this corresponded to a slightly different point reduction, which translated to a reduction with at least two points since the symptom scores are integer. Similarly, the clinically relevant reduction in the overall score was found to be nearby six points. Clinically relevant reductions of 2 points respectively 6 points were also found when the difference in mean symptom scores from “quite a bit” to “a bit” were considered.

All participants were asked to complete the MSQ before during and after the acupuncture treatment.

### Factors investigated for their relative importance

The factors investigated for their importance in obtaining a clinically relevant reduction in menopause-specific symptoms were all the variables that were collected from the baseline questionnaire completed by all women at inclusion into the ACOM study. This selection of factors was:

- Age, Chronic conditions, Body Mass Index
- Menopause-related factors: Menstruation last year, Duration of hot flushes, Number of births, Incontinence.
- Social factors: Level of education: Vocational, short and medium cycle higher education, long cycle higher education, and other (not specified education or don’t know). Employment: employed, unemployed, Household status: living alone, living with others, Smoking: yes, no, Alcohol use: no alcohol, ≤ 14 units per week, > 14 units per week, Physical activity: no, 1–3 times per week, ≥ 4 times per week, Previous experience with acupuncture: no, yes.
- Further, we included allocation status in the analysis.

### Statistical analysis

The relative importance of the above factors for each of the four menopause-specific subscales and for the overall score was assessed in a dominance analysis [15]. A dominance analysis attempts to distribute the explained variance of the full multivariable model into the parts attributable to the individual factors. In the present analyses, the model was a multivariable logistic regression model and its fit was assessed by McFadden’s pseudo R^2^. The relative importance of each factor was computed as the mean increase in the pseudo R^2^ value obtained by adding the factor to each possible model containing the remaining variables. The relative importances were then normalized such that they add up to 1. The implemented definition of relative importance explicitly accounts for correlation between factors.

To characterize the effect of the most important factors a multivariable logistic regression with the top three important factors for each of the outcomes was performed.

## Results

Mean age of the women were 54.8 years and on average, they experienced hot flushes for 4.1 years. Baseline data stratified by experienced clinical reduction in the four symptom scales and overall composite scale is presented in Table 1.

**Table 1:** Baseline data stratified by experienced clinical reduction in the four symptom scales and overall composite scale

	Total	Hot flushes Clinical reduction		Day-and-night sweats Clinical reduction		General sweating Clinical reduction		Menopausal-specific sleeping problems Clinical reduction		Overall Clinical reduction	
		Yes	No	Yes	No	Yes	No	Yes	No	Yes	No
Total, n	67	43	24	33	34	19	48	40	27	32	35
**Age, mean (sd)**	54.81 (4.53)	54.86 (4.03)	54.71 (5.41)	55.06 (4.17)	54.56 (4.91)	54.63 (4.54)	54.88 (4.58)	54.65 (4.24)	55.04 (5.01)	55.03 (4.46)	54.6 (4.65)
**Employment, n (%)**											
Employed	60 (89.55)	39 (90.7)	21 (87.5)	30 (90.91)	30 (88.24)	17 (89.47)	43 (89.58)	36 (90)	24 (88.89)	28 (87.5)	32 (91.43)
Unemployed	7 (10.45)	4 (9.3)	3 (12.5)	3 (9.09)	4 (11.76)	2 (10.53)	5 (10.42)	4 (10)	3 (11.11)	4 (12.5)	3 (8.57)
**Education, n (%)**											
Vocational	16 (23.88)	16 (37.21)	0 (0)	11 (33.33)	5 (14.71)	6 (31.58)	10 (20.83)	14 (35)	2 (7.41)	13 (40.63)	3 (8.57)
Short or medium cycle higher education	7 (10.45)	3 (6.98)	4 (16.67)	3 (9.09)	4 (11.76)	3 (15.79)	4 (8.33)	3 (7.5)	4 (14.81)	3 (9.38)	4 (11.43)
Long cycle higher education	35 (52.24)	19 (44.19)	16 (66.67)	14 (42.42)	21 (61.76)	8 (42.11)	27 (56.25)	17 (42.5)	18 (66.67)	11 (43.38)	24 (68.57)
Other	9 (13.43)	5 (11.63)	4 (16.67)	5 (15.15)	4 (11.76)	2 (10.53)	7 (14.58)	6 (15)	3 (11.11)	5 (15.63)	4 (11.43)
**Household**											
Living alone	5 (7.46)	4 (9.3)	1 (4.17)	2 (6.06)	3 (8.82)	1 (5.26)	4 (8.33)	5 (12.5)	0	3 (9.38)	2 (5.71)
Living with others	62 (92.54)	39 (90.7)	23 (95.83)	31 (93.94)	31 (91.18)	18 (94.74)	44 (91.67)	35 (87.5)	27 (100)	29 (90.63)	33 (94.29)
**Physical activity, n (%)**											
No physical activity	10 (14.93)	4 (9.3)	6 (25)	4 (12.12)	6 (17.65)	1 (5.26)	9 (18.75)	5 (12.5)	5 (18.52)	4 (12.5)	6 (17.14)
1–3 times per week	40 (59.7)	30 (69.77)	10 (41.67)	20 (60.61)	20 (58.82)	12 (63.16)	28 (58.33)	26 (65)	14 (51.85)	20 (62.5)	20 (57.14)
≥ 4 times per week	17 (25.37)	9 (20.93)	8 (33.33)	9 (27.27)	8 (23.53)	6 (31.58)	11 (22.92)	9 (22.5)	8 (29.63)	8 (25)	9 (25.71)
**Smoking, n (%)**											
Yes	3 (4.48)	2 (4.65)	1 (4.17)	2 (6.06)	1 (2.94)	0	3 (6.25)	2 (5)	1 (3.7)	2 (6.25)	1 (2.86)
No	64 (95.52)	41 (95.35)	23 (95.83)	31 (93.94)	33 (97.06)	19 (100)	45 (93.75)	38 (95)	26 (96.3)	30 (93.75)	34 (97.14)
**Alcohol, n (%)**											
No alcohol	9 (13.43)	9 (20.93)	0	5 (15.15)	4 (11.76)	3 (15.79)	6 (12.5)	8 (20)	1 (3.7)	6 (18.75)	3 (8.57)
≤ 14 unites per week	45 (67.16)	28 (65.12)	17 (70.83)	24 (72.73)	21 (61.76)	15 (78.95)	30 (62.5)	26 (65)	19 (70.37)	22 (68.75)	23 (65.71)
> 14 units per week	13 (19.4)	6 (13.95)	7 (29.17)	4 (12.12)	9 (26.47)	1 (5.26)	12 (25)	6 (15)	7 (25.93)	4 (12.5)	9 (25.71)
**Body mass index, mean (sd)**	25.21 (4.3)	25.51 (4.57)	24.67 (3.8)	25.05 (5.03)	25.36 (3.52)	25.07 (3.62)	25.26 (4.58)	25.45 (4.29)	24.84 (4.37)	25.54 (5.04)	24.9 (3.54)
**Menstruation in the last year, n (%)**											
Yes	18 (26.87)	12 (27.91)	6 (25)	10 (30.30)	8 (23.53)	7 (36.84)	11 (22.92)	11 (27.5)	7 (25.93)	8 (25)	10 (28.57)
No	49 (73.13)	31 (72.09)	18 (75)	23 (69.70)	26 (76.47)	12 (63.16)	37 (77.08)	29 (72.5)	20 (74.07)	24 (75)	25 (71.43)
**Number of births, n (%)**											
None	4 (5.97)	2 (4.65)	2 (8.33)	1 (3.03)	3 (8.82)	0	4 (8.33)	2 (5)	2 (7.41)	2 (6.25)	2 (5.71)
One	13 (19.4)	7 (16.28)	6 (25)	4 (12.12)	9 (26.47)	2 (10.53)	11 (22.92)	8 (20)	5 (18.52)	5 (15.63)	8 (22.86)
Two	36 (53.73)	24 (55.81)	12 (50)	19 (57.58)	17 (50)	14 (73.68)	22 (45.83)	21 (52.5)	15 (55.56)	18 (56.25)	18 (51.43)
More than two	14 (20.9)	10 (23.26)	4 (16.67)	9 (27.27)	5 (14.71)	3 (15.79)	11 (22.92)	9 (22.5)	5 (18.52)	7 (21.88)	7 (20)
**Urinary Incontinence, n (%)**											
No	23 (34.33)	17 (39.53)	6 (25)	15 (45.45)	8 (23.53)	4 (21.05)	19 (39.58)	14 (35)	9 (33.33)	14 (43.75)	9 (25.71)
Yes	44 (65.67)	26 (60.47)	18 (75)	18 (54.55)	26 (76.47)	15 (78.95)	29 (60.42)	26 (65)	18 (66.67)	18 (56.25)	26 (74.29)
**Chronic disease, n (%)**											
Yes	13 (19.4)	8 (18.6)	5 (20.83)	7 (21.21)	6 (17.65)	4 (21.05)	9 (18.75)	6 (15)	7 (25.93)	6 (18.75)	7 (20)
No	54 (80.6)	35 (81.4)	19 (79.17)	26 (78.79)	28 (82.35)	15 (78.95)	39 (81.25)	34 (85)	20 (74.07)	26 (81.25)	28 (80)
**Current use of alternative treatment, n (%)**											
Yes	50 (74.63)	31 (72.09)	19 (79.17)	23 (69.70)	27 (79.41)	16 (84.21)	34 (70.83)	26 (65.00)	24 (88.89)	23 (71.88)	27 (77.14)
No	17 (25.37)	12 (27.91)	5 (20.83)	10 (30.30)	7 (20.59)	3 (15.79)	14 (29.17)	14 (35.00)	3 (11.11)	9 (28.13)	8 (22.86)
**Duration of hot flushes, mean (sd)**	4.1 (3.48)	3.69 (3.53)	4.82 (3.35)	4.14 (4.13)	4.05 (2.78)	3.62 (3.65)	4.29 (3.44)	4.22 (3.76)	3.91 (3.08)	4.31 (4.12)	3.9 (2.82)
**Allocation status, n (%)**											
Control	31 (46.27)	17 (39.53)	14 (58.33)	16 (48.48)	15 (44.12)	9 (47.37)	22 (45.83)	16 (40)	15 (55.56)	14 (43.75)	17 (48.57)
Intervention	36 (53.73)	26 (60.47)	10 (41.67)	17 (51.52)	19 (55.88)	10 (52.63)	26 (54.17)	24 (60)	12 (44.44)	18 (56.25)	18 (51.43)

52 (77.6%) experienced a clinically relevant reduction in any of the four symptom scales, 48 (71.6%) in any of the VMS subscales, and 32 (47.8%) in the overall composite score, respectively.

Women with vocational education were most likely to experience improvement compared to women with longer education. Beyond level of education, other factors of some importance for clinically relevant reduction were no alcohol consumption, two or more births and urinary incontinence (Table 2).

**Table 2:** The effect of the three most important factors for each of the outcomes

	Hot Flushes (HF)		Day-and-Night Sweats (DNS)		General Sweating (GS)		Menopausal-Specific Sleeping Problems		Overall change in symptoms	
	OR (95%CI)	*p*-value	OR (95%CI)	p-value	OR (95%CI)	p-value	OR (95%CI)	p-value	OR (95%CI)	p-value
**Education**		**0.0005**		**0.2219**		**0.5169**		**0.0097**		**0.0129**
Vocational	∞		3.83 (1.01; 16.37)	0.0865	1.90 (0.46; 7.90)	0.3732	9.43 (2.01; 70.07)	0.0133	9.36 (2.36; 48.84)	0.0038
Short or medicum cycle higher education	0.56 (0.06; 3.86)	0.5852	1.45 (0.22; 9.28)	0.6971	2.71 (0.38; 20.56)	0.3275	0.30 (0.03; 2.34)	0.2787	1.80 (0.28; 11.13)	0.5315
Long cycle higher education	ref	–	ref		ref		ref		ref	
Other	0.66 (0.10; 3.91)	0.6568	2.71 (0.54; 14.74)	0.2373	0.65 (0.08; 3.79)	0.6616	1.65 (0.28; 10.64)	0.5894	2.20 (0.45; 11.39)	0.3388
**Physical activity**		**0.1914**								
No physical activity	0.33 (0.05; 1.69)	0.2170	–	–	–	–	–	–	–	–
1–3 times per week	ref		–	–	–	–	–	–	–	–
≥4 times per week	0.29 (0.06; 1.30)	0.1146	–	–	–	–	–	–	–	–
**Alcohol**		**0.0038**				**0.2178**		**0.0736**		**0.2298**
No alcohol	∞		–	–	1.27 (0.22; 6.70)	0.7839	8.14 (1.17; 166.28)	0.0973	2.13 (0.41; 12.74)	0.3884
≤14 units per week	ref		–	–	ref		ref		ref	
> 14 units per week	0.29 (0.05; 1.41)	0.1461	–	–	0.19 (0.01; 1.29)	0.1894	0.75 (0.16; 3.37)	0.7113	0.37 (0.06; 1.67)	0.2413
**Number of births**				**0.2176**		**0.2397**				
None	–	–	0.32 (0.01; 3.60)	0.4479	0.00		–	–	–	–
One	–	–	0.47 (0.10; 1.89)	0.3139	0.42 (0.06; 2.07)	0.3368	–	–	–	–
Two	–	–	ref		ref		–	–	–	–
More than two	–	–	2.38 (0.60; 10.61)	0.2367	0.62 (0.11; 2.91)	0.5672	–	–	–	–
**Urinary incontinence**				**0.1376**						**0.1582**
No	–	–	ref		–	–	–	–	ref	
Yes	–	–	2.00 (0.77; 7.27)	0.2262	–	–	–	–	2.31 (0.72; 7.85)	0.1695
**Current use of alternative treatment**								**0.0038**		
No	–	–	–	–	–	–	ref		–	–
Yes	–	–	–	–	–	–	0.12 (0.02; 0.53)	0.0112	–	–

## Discussion

Women with vocational education were most inclined to experience an improvement in menopausal symptoms while women with higher education to a lesser degree experienced improvement. Beyond level of education, other factors of some importance for clinically relevant reduction were no alcohol consumption, two or more births and urinary incontinence.

Around 12% of Danish women between 45 and 64 years stated that they had used acupuncture within the last year [16]. Women with longer education were less likely to use acupuncture. This may be due to a more critical perception towards acupuncture or a lower expectation towards the efficacy of acupuncture. Perception of and expectations has been identified as central to the perceived efficacy of acupuncture analgesia [17] and we hypothesize that the same mechanisms may explain our findings. However, we do find that current use of alternative medication actually decrease the effect acupuncture in the sleeping dimension, but not in any other dimensions.

A moderate consumption of alcohol has been associated with delayed menopausal onset [18], which may be due to an estrogenic effect caused by alcohol [19]. The mechanisms behind our findings were difficult to explain based on the design of our study. In order to study these mechanisms we suggest conducting research to explore perceptions and experiences of acupuncture for women with moderate to severe menopausal symptoms.

Previous studies on the effect of acupuncture treatment for menopausal symptoms have typically focused on VMS and reported a 30–60% reduction in VMS frequency among women receiving treatment [20–25]. This is in line with our findings on the composite score (48%). However, these studies report aggregated data and consequently do not identify factors associated with a perceived clinically relevant effect. Further, the outcomes used are not obtained from validated scales and the interventions are characterized by individualized acupuncture modalities.

Only one study has surveyed the probability for a reduction in VMS over 8 weeks: A small group (11%) experienced an 80% reduction in VMS, 59% reported a 40% reduction and 41% did not experience a reduction in VMS frequency at 8 weeks [12]. In our study 48 (72%) reported a clinically relevant reduction in any of the three VMS subscales. It is difficult directly to compare our results due to our use of a validated outcome and our definition of a clinically relevant outcome [9]. However, based on our study we found that the majority of women actually benefitted from the acupuncture treatment in one or more of the VMS dimensions we surveyed. Consequently, we suggest that the larger effect we find compared to previously reported effects may be due to our use of the rigorous validated outcome measure aimed to identify bothersome menopausal symptoms.

Our definition of a clinically relevant reduction was based on the data used for the validation of the MSQ. A clinically relevant reduction for the overall composite score was defined as a reduction of 6 points. This outcome, while not unidimensional, is potentially more sensitive. For detailed information on the MSQ please refer to the validation article [9].

The present study had a high participant adherence and used valid outcome measures with 100% response rate. Further, the intervention was found to be well-tolerated by the participants with only few mild side-effects and manageable for general practitioners. We used a brief and standardized acupuncture modality in order to evaluate the effectiveness and the determinants for a clinically relevant reduction in symptoms. Consequently, it may be transferred into most clinical settings, even outside of Denmark, due to the high external validity of the study. This standardized modality may be seen as a weakness for some, but was well accepted by the general practitioners who were doing the acupuncture [10].

The study was not powered to find clinical relevant reductions. However, significant results have a < 5% chance for a false positive result regardless of sample size. Due to the power in the present study some factors that may have clinically importance could not be detected. We excluded women who participated in other interventions for menopausal symptoms in order to minimize the influence of treatment effects of co-interventions, which makes the interpretation of the surveyed effects as caused by the acupuncture treatment more pertinent.

In this study we used WMA theory, in which acupuncture predominantly is understood by stimulating the nervous system [13, 26]. The ovaries and uterus are innervated by sympathetic nerves from the spinal cords segment Th11 to S3 and the parasympathetic nerves from the segment S2 to S4. Our acupuncture modality are all placed within these segments [26]. Furthermore, acupuncture has been shown to stimulate beta-endorphins, serotonin and norepinephrine [26, 27], which are all believed to be related to temperature regulation and the generation of hot flushes [5]. However, the underlying mechanism of acupuncture still remains unknown, and it is likely that other acupuncture modalities also would find clinically relevant symptom reductions.

## Conclusions

Women with vocational education were most likely to experience an improvement in their menopausal symptoms compared to women with higher education. Beyond level of education, other factors of some importance for clinically relevant reduction were no alcohol consumption, two or more births and urinary incontinence. The mechanisms are poorly understood and we recommend further research in order to qualify recommendations to clinicians.

## Data Availability

Anonymized data can be made available upon reasonable request.

## Abbreviations

HF: Hot flushes
DNS: Day and night sweats
GS: General sweating
MSSP: Menopausal-specific sleeping problems
VSM: Vasomotor symptoms (VMS)
WMA: Western medical acupuncture
